# Innovative Pressure Sensor Platform and Its Integration with an End-User Application

**DOI:** 10.3390/s140610273

**Published:** 2014-06-11

**Authors:** Antonio Flores-Caballero, Dorin Copaci, María Dolores Blanco, Luis Moreno, Jaime Herrán, Iván Fernández, Estíbaliz Ochoteco, German Cabañero, Hans Grande

**Affiliations:** 1 Universidad Carlos III de Madrid, Avda. Universidad 30, 28911 Leganés, Madrid, Spain; E-Mails: dcopaci@ing.uc3m.es (D.C.); dblanco@ing.uc3m.es (M.D.B.); moreno@ing.uc3m.es (L.M.); 2 IK4-Cidetec, P*^o^* Miramón 196, 20009 San Sebastián, Spain; E-Mails: jherran@cidetec.es (J.H.); ifernandez@cidetec.es (I.F.); eochoteco@cidetec.es (E.O.); gcabanero@cidetec.es (G.C.); hgrande@cidetec.es (H.G.)

**Keywords:** systems integration, pressure sensor, bendable sensor, Rapid Control Prototyping, embedded software, end-user application

## Abstract

This paper describes the fully integration of an innovative and low-cost pressure sensor sheet based on a bendable and printed electronics technology. All integration stages are covered, from most low-level functional system, like physical analog sensor data acquisition, followed by embedded data processing, to end user interactive visual application. Data acquisition embedded software and hardware was developed using a Rapid Control Prototyping (RCP). Finally, after first electronic prototype successful testing, a Taylor-made electronics was developed, reducing electronics volume to 3.5 cm × 6 cm × 2 cm with a maximum power consumption of 765 mW for both electronics and pressure sensor sheet.

## 1. Introduction

Actual sensor system manufacturing and research are focused on producing lower-cost, simplified sensors and easier electronics integration, providing the same or enhanced features compared to earlier produced sensors. Furthermore, an important feature that is gaining more relevance is providing sensors that could be used in extremely harsh environments and requiring less quality power sources, non-laboratory-ready or professional ones. In this respect, easy-to-power systems using only a single 5 Volts or 3 Volts are clearly preferred.

Accordingly to these desired features that have been explained, an entire pressure sensor system is presented in this article; using an innovative design process that allows the production of bendable sensors in an easier and cheaper way than before and using a fast-to-use, fast-to-integrate electronics system that minimizes the typical huge amount of time required for system integration to only a couple of days. Composed in this way, an economically affordable pressure sensor system can be capable of working in dusty environments, requiring a single power source from a typical universal serial bus (USB) computer port.

The article is structured in a comprehensive manner. It starts with intensive explanations about the involved technologies, from the sensors to the electronics integration. It continues with comparisons and research aspects about similar available sensor and control electronics devices. Finally, it presents a discussion about the results and the entire system reliability.

## 2. Involved Technologies

A large concurrency of multidisciplinary technicians and experts were involved in the entire development process of the presented pressure sensor platform system, from experts in physics and chemistry and also electronic signal conditioning technicians to experts in real-time embedded systems and graphical programming applications. Due to this multidisciplinary nature, the different technologies can be easily distinguished.

### 2.1. Pressure Sensor Technology

**Figure 1.**
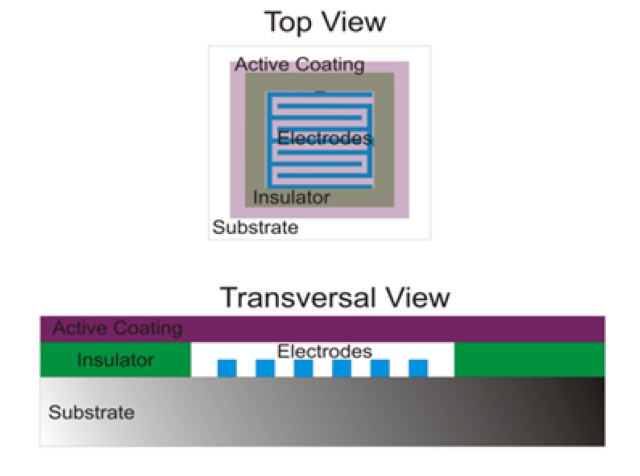
Scheme (top and transversal view) of one single sensor.

**Figure 2.**
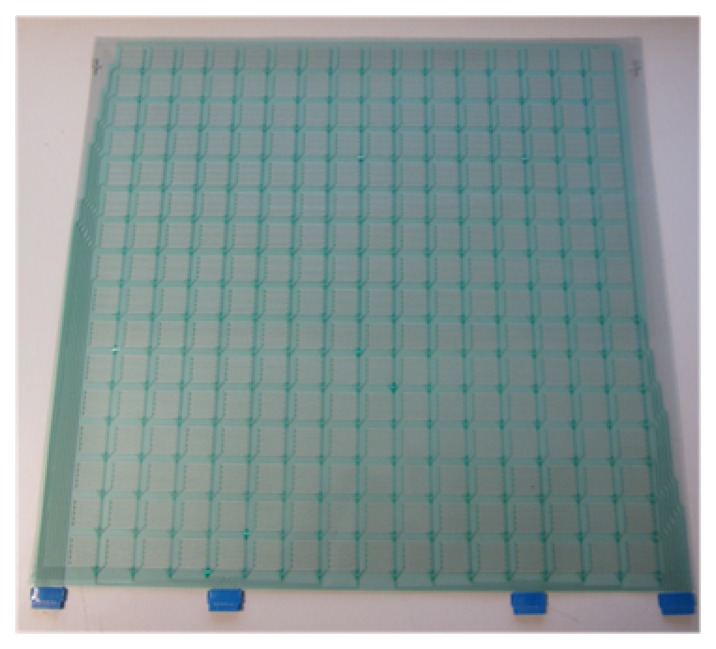
Picture of the 16 × 16 sensor array (45 cm^2^ × 45 cm^2^).

The pressure sensor sheet consists of a 256 analog sensor matrix, based on flexible printing electronics. The sensor configuration is formed by a 16 × 16 array with a spatial resolution of 2 cm^2^ of the tactile unit and a total area of 45 cm^2^ × 45 cm^2^. Each single sensor is formed by silver interdigitated electrodes defined by screen-printing technology on a polyethylene terephthalate (PET) flexible substrate and a poly(3,4-dioxythiophene) (PEDOT) conducting polymer on the top side of the electrodes acting as an active coating. An insulating acrylate elastomeric coating is used as a separator between the electrodes and the electroactive coating. Figure 1 shows a scheme of the sensor array (top and transversal view), and Figure 2 shows a picture of the 16 × 16 sensor array (45 cm^2^ × 45 cm^2^, there are a small separation between sensors).

The pressure sensor sheet construction, in a relatively simplistic way, comprises two stages. The first one is based on the well-known screen printing technology, for resistive path (electrodes at Figure 1) and insulator part creation over a flexible substrate. The second one represents the innovative feature of these presented sensors, the fabrication of the electroactive coating, placed over the electrodes at insulator fixed heights. These sensors are not based on dual contact similar electrodes; they are based on electrode to electroactive coating contact, making them easier, faster and cheaper to manufacture. This concept brings immunity to the misaligned problems related to prior electrode to electrode-based sensors. The electroactive coating does not require the typical painstaking screen printing procedures, like previous resistive paths. Furthermore, the electronics integration stage is remarkable easier due to the minor number of electric contacts.

The fabrication process of the electroactive coating is as follows: poly(3,4 ethylenedioxythiophene) (PEDOT) aqueous dispersions were prepared by using an ultrasonic processor (model UP 400 S from Hielscher GmbH) during the synthesis. As an example, ethylenedioxythiophene (1 mL, 9.4 mmol) and 3.5 g of poly(styrene sulfonate) sodium salt were dissolved in 100 mL of distilled water. To this mixture, an equimolar amount of ammonium peroxydisulfate (6.58 g, 28.8 mmol) dissolved in 50 mL of water was added dropwise over a period of 4 min. After 1 hour of reaction under ultrasonic irradiation, a dark blue PEDOT aqueous dispersion was obtained. The conducting flexible sheets were prepared by spin coating using a PET (polyethylene terephthalate, 5 cm^2^ × 5 cm^2^ in surface, 125 *μ*m in thickness) plastic sheet. Fifty milliliters of conducting polymer ink were dispensed on it, and it was rotated at 1500 rpm. A thin conducting film was created on the plastic sheet, giving a conducting flexible plastic sheet. The conductivity of the coating is 1.8 × 10^−3^ S/cm [1].

#### 2.1.1. Sensing Mechanism

The physical behavior of this pressure sensor can be easily understood if the surface relation between thlevel of abstraction for embedded software development for this e contact of the polymer and the electrodes is studied. As the contact area among the polymer and the electrodes increases, the resistance shows a lower value, due to the existence of additional parallel resistive paths. In this context, a smaller resistance sensor value is related to a higher Vout slope, according to the sensor signal conditioning configuration; this signal conditioning is detailed at Section 3.1. Anyway, as a strategic alternative and according to the application scenario, the sensor output response can be customized by tuning the electronics and/or the conducting polymer coating [1,2]. The signal conditioning circuitry, the insulator, as well the active coating thickness (see Figure 1 for better understanding) and the conductivity of the polymer coating (in this case, 1.8 × 10^−3^ S/cm) are related to the offset and the slope of the sensor response for each desired design, for each desired pressure measure range. In this article, 20-kg maximum pressure (20 kg per a spatial resolution of 2 cm^2^, a pressure of 10 kg/cm^2^ or 980.7 kPa) labeled sensors are being used.

According to the signal conditioning circuitry, explained in Section 3.1, due to the absence of any capacitive and inductive parts, the sensor bandwidth is only limited by the capabilities of the involved operational amplifiers. For stabilization purposes, a wait time slice of 0.5 ms is spent between each sensor row measurement; obviously, this wait time depends upon the operational amplifier quality; for state-of-the-art ones (and rather more expensive ones), a wait time of 0.1 ms could be more than enough. These technical aspects are taken into account at Section 3.2.

#### 2.1.2. Technologies on the Market

In the last few years, the development of pressure sensors has been based on different technologies and working principles: piezoresistive, based on dual screen printing resistive paths; capacitive sensors based on MEMS (micro-electronic-mechanical sensors) [3–5], capacitive based ones using up to 4 printed layers [6], polymer technology [7–9] and optical-reflexive and strain gauges based [10]. Optical-reflexive devices are extremely expensive and cannot be detached from their installation place, and strain gauges based sensors require larger signal conditioning electronics, mainly based on numerous expensive high-resolution differential analog-to-digital converters. Several companies have also commercialized these kinds of sensors [11–13], but none of these previous technologies relies on fabrication methods cheaper than the one shown in this article, a concept that leads to an easier fabrication process using only 2 layers, electroactive layer and electrodes layer, and easier integration electronics, due to the reduced number of electric contacts compared to previous ones. However, and despite the advances of the last few years, it is still not possible to develop a standard or universal pressure measuring system, due to the incompatibility among the different technologies, applications, integration capabilities, and sensing principles.

### 2.2. Electronics Technologies

The bendable pressure platform system utilizes a state-of-the-art 32-bit microcontroller (MCU) as the hardware interface between the analog sensor matrix and the personal computer (PC) visual application or other industrial-based digital interfaces.

Traditionally, the development of the embedded software, also called firmware, comprises many processes, such as the selection and design of the embedded controller, programming (usually via hand-written languages) the MCU, running and testing the program, as well as the verification of the required execution time and its performance. All these embedded controller design stages need to be restarted each time an error or malfunction is detected, making the implementation of really large and complex firmware pretty hard, consuming enormous amounts of time and money [14].

In the development of the embedded firmware that provides total control, data acquisition and processing for this presented system, a methodology based on the usage of a rapid control prototyping (RCP) software/hardware tool is used. Using an RCP enables a great set of interesting features; all the previously mentioned stages for firmware development can be accomplished using the same unified hardware platform, making possible as many test and iterations as engineers require in little time. Usually, an RCP system provides a higher level of abstraction, thanks to the usage of graphical programming languages, due to the complex nature of many input/output (I/O) analog and digital interfaces that can be obviated, making the use of an RCP system possible for a multidisciplinary audience [15,16], allowing them the easy integration of multiple analog, digital and communication interfaces at the same time for a standalone controller.

**Figure 3.**
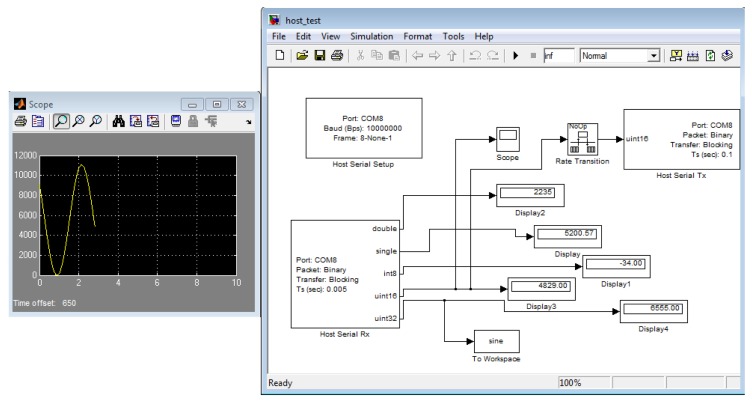
The Simulink® graphical programming environment, a simple data acquisition program, reading and storing data received from an STM32F4 target via universal serial bus (USB).

The RCP system in use for the work presented in this article is an in-house development of the University Carlos III of Madrid (UC3M), at RoboticsLab. The usage of any commercial available RCP system, such as dSPACE® (dSPACE®, Paderborn, Germany), xPC Target® (MathWorks®, Natick, MA, USA) [17], RT-Lab® (Opal-RT®, Montreal, Canada) or NI CompactRio/PXI® (National Instruments®, Austin, TX, USA), that represents the most popular RCP systems for control in automotive, industrial and aeronautic companies require a huge investment that many researchers cannot afford; the RCP solution adopted by the UC3M Robotics Lab is based on a system-on-chip (SOC) embedded system, which consists of a state-of-the-art, 32-bit MCU (STM32F407 and STM32F429 families from ST Microelectronics® manufacturer (STMicroelectronics®, Geneva, Italy) that provides plenty of digital and analog interfaces requiring no external add-on hardware. The SOC RCP-based solution proves itself to be the most competitive prototyping solution for both cost and performance [15,18,19]. The computational power of the UC3M RoboticsLab RCP system, a 32-bit MCU running at 168-180 MHz with floating point support via hardware, is almost double that required for more than 50% of industrial production market-ready control devices, which commonly require a 16–32-bit MCU running at 10-99 Mhz with no floating point hardware support; and no more than 25% require clock speeds far beyond 100 MHz [20,21]. These features and the small footprint size and minimal power consumption make the described UC3M RCP system ideal for laboratory prototyping, as well as for final hardware implementation, both stages accomplished as a single development stage. The high level of abstraction for embedded software development for this described RCP system is provided by the usage of MATLAB/Simulink® (MathWorks®), which provides a graphical programming language and is ideal to develop control systems. As Figure 3 shows, the MATLAB/Simulink® programming environment is graphically based on simple appearance blocks that implement different features, like I/O access.

Each block can be interpreted as a configurable, modular piece of code, each block being completely tested and debugged during its implementation, ensuring that bigger systems based on these modules will work flawlessly. For this purpose, the proposed RCP system requires the installation of additional software into the MATLAB/Simulink® Library browser; this software addition consist of two custom developed block sets, requiring no additional commercial software, like Embedder Coder® [15,18] or xPC Target® [17]. The first block set works as the base software; it is provided by a third party company called Aimagin Ltd. (Aimagin®, Bangkok, Thailand) and is free, covering many I/O peripherals. The second of these block sets, which works as an extension of the previous one, has been entirely developed by UC3M RoboticsLab and is the core of the proposed RCP system, providing high speed universal serial bus (USB) data exchange links with MATLAB/Simulink® and advanced features, like real-time multitasking support, complete I/O peripheral support and some very specialized performance optimizations to the generated code.

**Figure 4.**
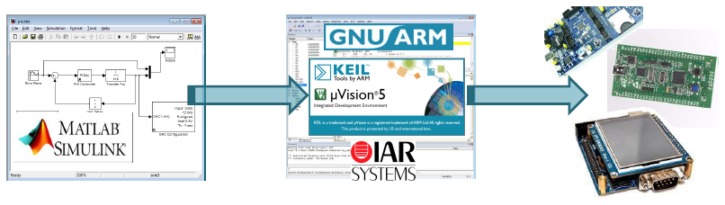
The Simulink® modeling workflow using the University Carlos III of Madrid (UC3M) rapid control prototyping (RCP) system. From left to right, Simulink generates all the required source code for the selected compiler and automatically loads the resulting firmware into the microcontroller (MCU).

Once the needed software for the RCP system is installed, the user only needs to develop their firmware using Simulink® and MATLAB® scripting, if needed; after this, the RCP hardware needs to be connected directly to a USB port of the PC computer, and like the last required action, the user needs to do a single “mouse click” in the Simulink® build model button. The source code will be generated, compiled and loaded into the MCU automatically. Figure 4 illustrates the firmware development stages. Source code generation and compiling is designed to use one of these three different compiler environments, the free one, GNU-ARM , and the commercial ones, Keil Uvision® (ARM Germany®, Grassbrunn, Germany) and IAR Ewarm® (IAR Systems AB®, Uppsala, Sweden).

Thanks to this RCP system, the embedded software for the pressure platform system and all testing stages was accomplished in only one working day.

## 3. Integration Stages

**Figure 5.**
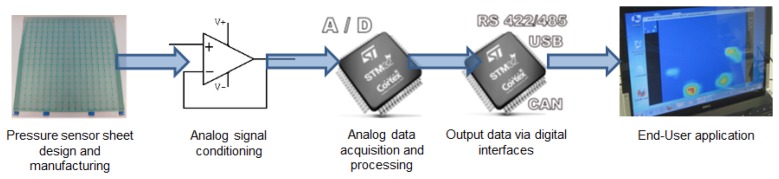
Integration stages, from pressure sensor sheet to an useful system.

The bendable pressure platform system comprises a series of interconnected individually parts or stages. Figure 5 shows all the integration stages and their relationships. Traditionally, the stages named “analog data acquisition and processing” and “output data via digital interfaces” take several weeks to be accomplished, using hand-written languages by experts in embedded architectures. However, due to the usage of the advanced RCP system provided by the UC3M RoboticsLab, these two stages are implemented and fully tested in a single day. Due to the usage of mass production, market-available hardware, the resulting control electronics reach the final end-user format immediately.

### 3.1. Analog Signal Conditioning

**Figure 6.**
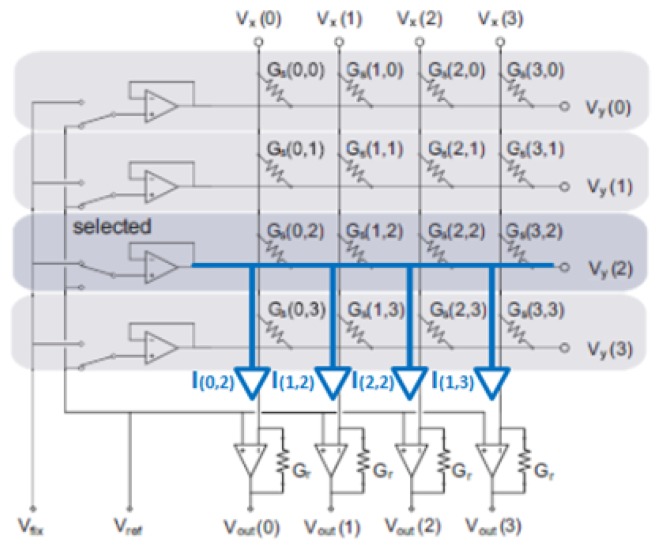
Anti-cross-talking signal conditioning electronic circuit.

One of the most common drawbacks for piezoresistive matricial sensors for multi-touch applications is the cross-talk effects, due to parasitic resistive paths. In this way, compensating techniques are required to minimize these effects. A few strategies to reduce the interference caused by these resistive paths have been proposed [22], although the best one that is commonly implemented is grounding/virtual grounding. Its goal consists in having the same voltage at both sides of the parasitic resistors, so that they are virtually short-circuited. The circuitry in the Figure 6 shows how to reduce the undesired contribution of cross-talk on the measuring circuit [23].

In this circuit (see Figure 6 for the referenced terms), one row (k) is selected by an analog switch, and a fixed voltage (*V_fix_*) is given by a buffer. The other rows are kept at the reference voltage.

The detection circuit consists of buffered analog switches and operational amplifiers in a transimpedance configuration. Buffers and transimpedance amplifiers keep column and row voltages constant independently of the actual sensor array's impedance. In order to keep this active cross-talking compensation functionality, operational amplifiers were carefully selected. The slew rate, bandwidth and current output requirements are determinant in this circuit, to avoid operational saturation and, also, circuit malfunction.

Applying Ohm's law, the current of each sensor element can be described as seen in Equation (1):

(1) $$I(i,j)=G_{s}(i,j)V_{s}(i,j)$$

(2) $$V_{y}(i=k)=V_{\text{fix}}$$

(3) $$V_{y}(i\neq k)=V_{\text{ref}}$$

(4) $$V_{x}(i)=V_{\text{ref}}$$

As *V s*(*i, j*) = *V x*(*i*) − *V y*(*j*), then the initial equation can be transform as following:

(5) $$I(i,j=k)=G_{s}(i,j)(V_{\text{fix}}-V_{\text{ref}})$$

(6) $$I(i,j\neq k)=G_{s}(i,j)(V_{\text{ref}}-V_{\text{ref}})=0$$

Therefore, parasitic impedances are virtually grounded, as they are kept at the same voltage on both sensor electrodes. The measurement current flow over each column can be described by Equation (7) according to Kirchhoff's current law:

(7) $$I(i,k)=(V_{\text{fix}}-V_{\text{ref}})G_{s}(i,k)=(V_{\text{out}}-V_{\text{ref}})(i,k)G_{\gamma }$$

(8) $$G_{s}(i,k)=\frac{\left(V_{\text{out}}-V_{\text{ref}})(i,k\right)}{\left(V_{\text{fix}}-V_{\text{ref}}\right)}G_{\gamma }$$

Equation (8) shows that sensor conductance, *G_s_*(*i, k*), can be calculated from the output voltage of the operational amplifier, *V_out_* (*i, k*), and a gain determinate by *G*_γ_ and the difference of tension *V_fix_* − *V_ref_*. By scanning (*k* = 0, 1, 2, ), all *G_s_*(*i, j*) can be measured. In impedance terms, the output voltages are calculated, as Equation (9) shows the linear output and its inversely proportional relationship of *V_out_* − *V_ref_* with the sensor's resistance:

(9) $$\left(V_{\text{out}}-V_{\text{ref}})=\frac{G_{s}(i,k)}{G_{\gamma }}(V_{\text{fix}}-V_{\text{ref}})=\frac{R_{\gamma }}{R_{s}(i,k)}(V_{\text{fix}}-V_{\text{ref}}\right)$$

### 3.2. Analog Data Acquisition and Processing

The analog data provided by the pressure sensors are read in row groups. Each row includes 16 analog output sensors; each sensor provides an output voltage from one Volt to three Volts, inversely proportional to its impedance; lower impedances produce greater voltages. These 16 sensors per row are read at the same time by the 16 analog-to-digital input converters of the RCP system. Each entire row is converted into digital values 10 times, in order to apply low-pass filtering; this operation is done within a time slice of one millisecond. When a row sensor datum is captured and processed, the RCP system changes its digital outputs to select the next row; these digital outputs are connected to the signal conditioning electronics and serve to select what row needs to be read. In order to provide stabilization to the conditioning electronics, a time slice of about 0.5 milliseconds is left from row selection until analog row reading.

When the last row is reached, read and processed, the resting time of the 0.5 millisecond time slice is occupied by the center of gravity (COG) calculations, which take minimal computational resources for this RCP system. After COG calculations, the analog matrix data and the COG are sent via many digital interfaces at the same time, in the same 0.5 millisecond time slice.

Like the bendable pressure sensor sheet consisting of a 16 × 16 analog sensor matrix and since each row data acquisition and treatment takes one millisecond, the update refresh rate for the entire analog matrix data and COG is 62.5 Hz.

### 3.3. Output Data via Digital Interfaces

The obtained analog matrix data and COG are sent when the last analog sensor row is read and filtered and after the COG calculations; there are various digital interfaces that allow the direct sending of these data in only one data packet. These digital interfaces are the Complementary Metal-Oxide-Semiconductor (CMOS) level (from 0 to 3.3 Volts) universal asynchronous receiver-transmitter (UART) running at six megabits/s (Mbit/s), the RS-422/485-level (intended for industrial usage) UART running at 6 Mbit/s and the universal serial bus (USB) running at a selectable speed of 12 or 480 Mbit/s.

**Figure 7.**
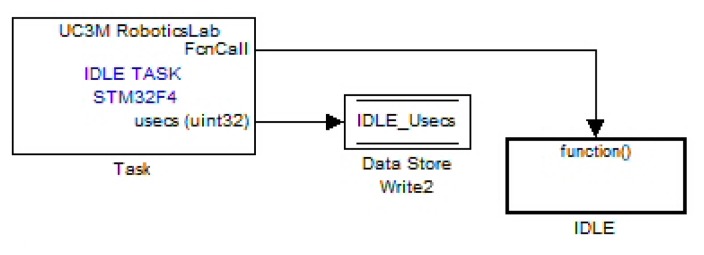
The idle task block enables the usage of the available free time of the MCU; this block continuously executes the connected subsystem when the higher preemptive priority task ends.

**Figure 8.**
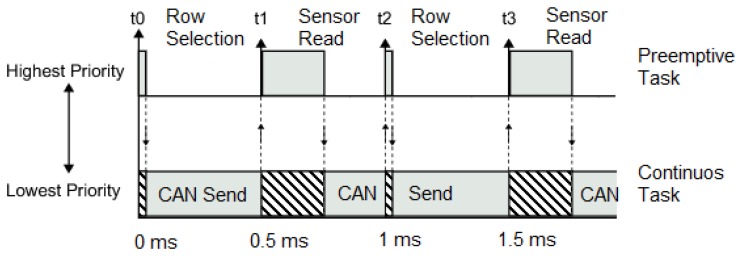
The idle task block enables a real-time multitasking environment without needing a real-time operating system; this figure shows the tasks execution over time.

The case of the Controller Area Network (CAN) bus 2.0 B (running at its maximum bandwidth of 1 Mbit/s) interface is different. This bus specification does not allow the transmission of huge data messages. The maximum data length for each CAN bus transmitted message is eight bytes. The transmission of the 256 analog sensor data (each of them consisting of two data bytes) and the COG data requires 65 CAN bus messages. These messages are sent without disturbing the preemptive real-time task that refreshes the analog matrix data and COG value, thanks to an advanced real-time multitasking feature of the UC3M RCP system that allows the usage of free available computational time after preemptive task completion, a feature that is not available in the Simulink® default code generation templates. The preemptive task, the higher priority task, is the analog sensor matrix data acquisition and processing; Figure 7 shows the Simulink® block of the UC3M add-on block set that implements this useful feature, and Figure 8 illustrates graphically how these explained tasks are being executed over time by the UC3M RCP system.

### 3.4. End-User Application

The bendable pressure platform system end-user application is a totally graphical based one. The application's goal is to provide an on-screen color map pressure representation and display the center of gravity (COG) over the same color map. It is intended to be used independently of common scientific data analysis and algorithm software packages, like LabView® (National Instruments®, Austin, TX, USA) or MATLAB®. Due to the fact that the performance is the key for graphical based software, standalone PC computer applications made with these software packages usually perform at low frame rates. The programming language selected for this application is the C language, the best performance only can be obtained using compiled languages that provide pure machine code, instead of using interpreted or dynamically recompiled languages, such as Java or Python.

The end-user application receives the analog matrix data and COG via the USB connection or via other industrial-based communication interfaces, like RS-422/485, that work at the same time in the RCP system. However, nowadays, if the application needs to be run on a PC-style computer, the USB connection is the best and most affordable option.

The end-user application shows the received analog matrix data in the PC computer screen in an interpolated way. This interpolation is based in a custom parallelizable bilinear filtering computation. Two versions of the end-user application software are implemented. The first version is based on pure software image rendering, optimized for dual-core or hyper-threaded (HT®) processors. This relatively primitive version obtains a 45% better performance for any dual-core processor and a 15% benefit for single-core HT processors. A single-core processor experiences no adverse performance impact executing this software-rendered version. Furthermore, a three- and four-thread (for three- or four-core enabled processors) intended version was tested, and severe adverse impact performance was observed for HT processors and single-core processors. Due to these bad performances, only the dual thread software-rendered version can be used. Like statistical data, the dual core version of the software, running on Intel Core 2 Duo P9600® (Intel®, Santa Clara, CA, USA) running at 2.66 Ghz, requires 24 ms to render one interpolated image, and an Intel Pentium 4 HT Extreme Edition® running at 3.2 Ghz requires 65 ms. This software rendered version uses DirectX® DirectDraw® (Microsoft Corporation®, Redmond, WA, USA) to paint directly into the video frame buffer for better performance purposes, but requires no hardware acceleration at all.

Another version of the same end-user application was developed, a version that nowadays requires basic video hardware acceleration capabilities. This hardware-rendered version uses DirectX® (exactly Direct3D®) or OpenGL (SGI®, Milpitas, CA, USA) application programming interfaces (API) and uses hardware-enabled functions to interpolate the image, using bilinear filtering; this version is easier to implement, and the needed time to render one interpolated image is minimal, also for extremely low-end computers with basic video hardware capabilities.

## 4. Experimental Results

### 4.1. Single Sensor Results

**Figure 9.**
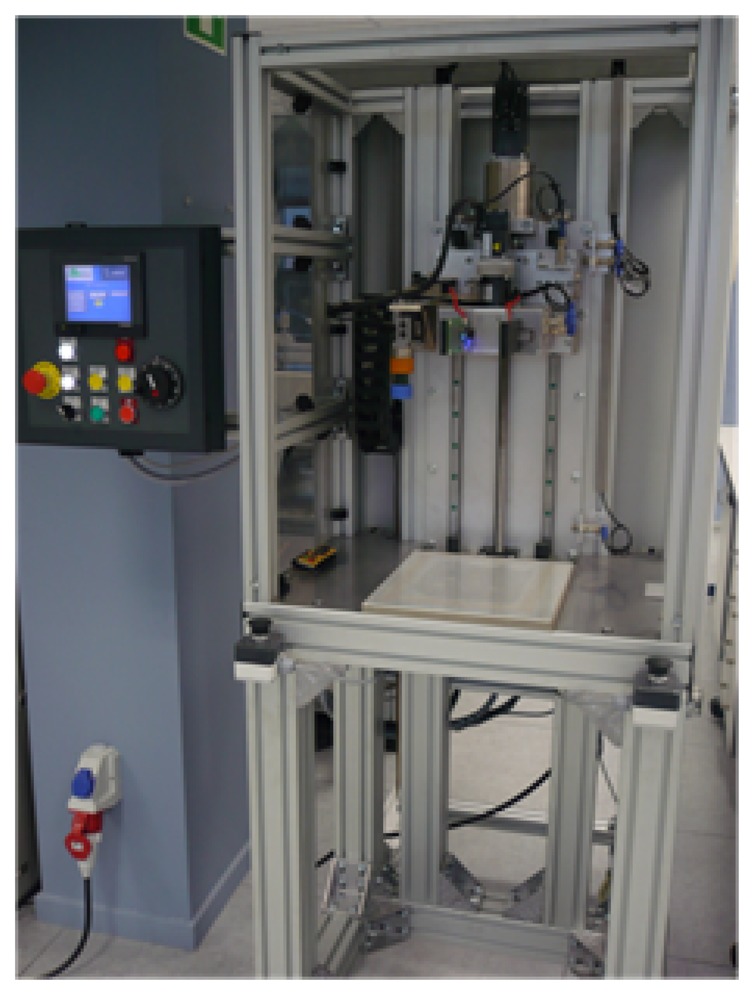
Load cell pressure characterization platform.

Electrical characterization was performed by a custom designed automatic platform. Figure 9 shows a photograph. The system consists of two parts: the first part is a fixed base optimized to place the sensor, and the second one is a *Y*-axis dynamic platform controlled by a Mitsubishi HF-KP43B (Mitsubishi®, Tokyo, Japan) servo motor of 400 W with an electromagnetic brake in charge of applying the force (pressure). A load cell of 75 kg and an amplifier, to convert the applied force in an electrical signal, are integrated in the platform. Moreover, a programmable logic controller (PLC) (FX3U-48M) from Mitsubishi controls the characterization system.

The load cell, as well as the pressure sensor output data are acquired using the same signal conditioning system, described previously in this article in Section 3.1, and low-cost, 12-bit analog to digital (A/D) converters, due to these sensors being intended to be used via low-cost electronics integration systems. Finally, the data are transmitted to a PC in real time through RS-232. The data is transmitted in their raw format, without any type of data processing. The intention is to check if the sensor itself contains a non-desired hysteresis effect.

**Figure 10.**
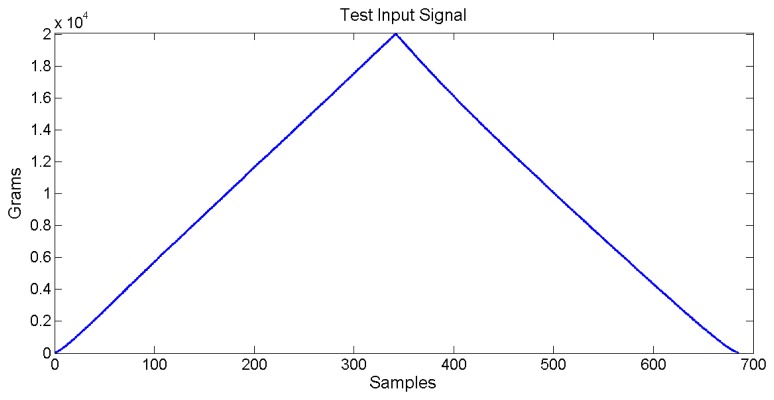
Input signal applied to the pressure sensor at characterization.

The sensor used in these characterization tests is a single sensor of 2 cm^2^ designed for a maximum measurement of 20 kg, the same type used for the pressure sensor sheet. The characterization machine does not provide enough surface to test a large sensor matrix directly. The input signal is a triangle based one. The loading stage goes from 0 to 20,000 g. The unloading stage returns from 20,000 to 0 g, the total time for each load/unload iteration is six seconds. The maximum allowed sensor output is 3 V, limited by A/D capabilities. *V_ref_* is equal to 1 V, which is the minimum output response. Figure 10 shows the input signal applied to the pressure sensor.

**Figure 11.**
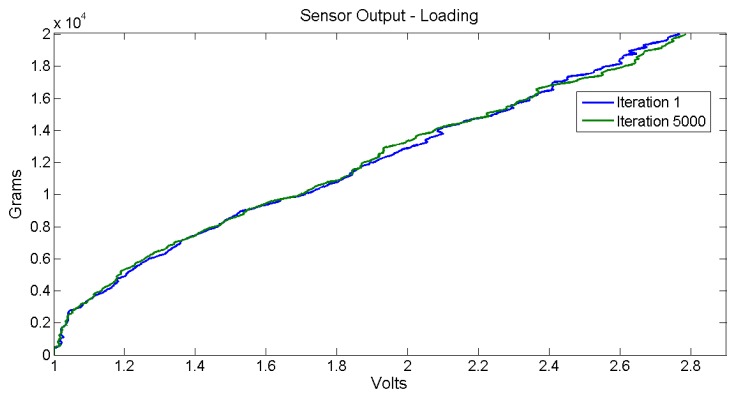
Raw sensor output response comparison for the loading stage.

The number of iterations reached up to 5000. The next figures illustrate the raw data acquired from the sensor and the signal conditioning system. Figure 11 shows the raw sensor output response for the loading stage at first and last iteration.

**Figure 12.**
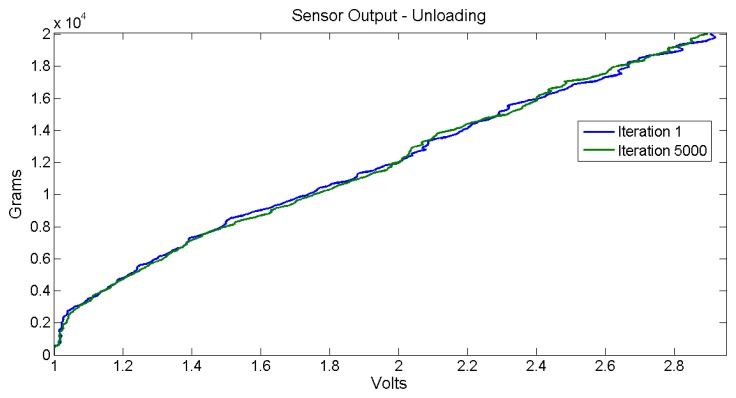
Raw sensor output response comparison for the unloading stages.

Figure 12 illustrates a comparison response for the unloading stage for the first iteration and the last one.

**Figure 13.**
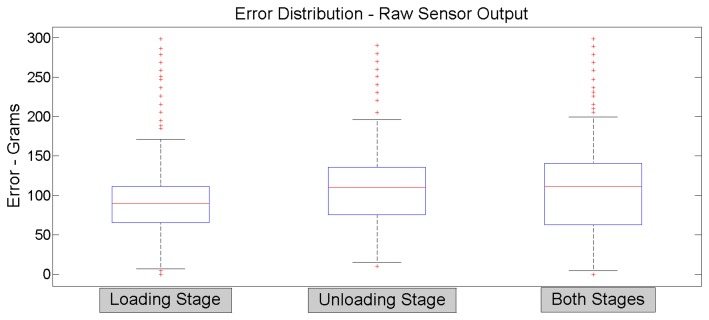
Raw sensor output error distribution.

Obviously, the illustrated sensor output data comes from direct acquisition, without any type of filtering or oversampling features. These are raw data that correspond to a single sensor output per input signal sample. These graphs show that the sensor response presents no noticeable hysteresis effect; also, the sensor output behavior does not present major changes after a thousand uses. The aw sensor data output error is found between 15 and 300 g (a maximum error of 1.5%). The objective is to develop a real-time data processing in the embedded electronics to maintain the maximum measured error near the lowest raw error value. In order to minimize actual disturbances, an oversampling of 10 sensor output samples and a simple low-pass filtering is recommended. This data processing technique is used for measuring each sensor output of the pressure sheet with noticeably improved results compared to raw measuring. The next section provides more information about the measuring aspects. Each graph, Figures 11 and 12, presents clearly two linear zones; their corresponding slopes are different. The first linear zone goes from 0 to 250 g; this zone presents extremely poor resolution and is not recommended to be used. The second linear zone goes from 250 g to 20 kg and appears pretty much linear, at least being easy to be mathematically modeled. This last one is the suggested usage zone. Figure 13 illustrates the error measurement distribution for loading, unloading and both stages for the 5,000 iteration test for the raw sensor output.

### 4.2. Pressure Sensor Sheet Results

Due to the low amount of time spent on the development stages, thanks to the expertise in embedded design using the RCP system, the great knowledge about graphical programming and the great experience in signal conditioning of the entire team involved in the development of the pressure platform system, several tests could be accomplished in the system that led to the system's performance improvements and quick, simple failure detections.

**Figure 14.**
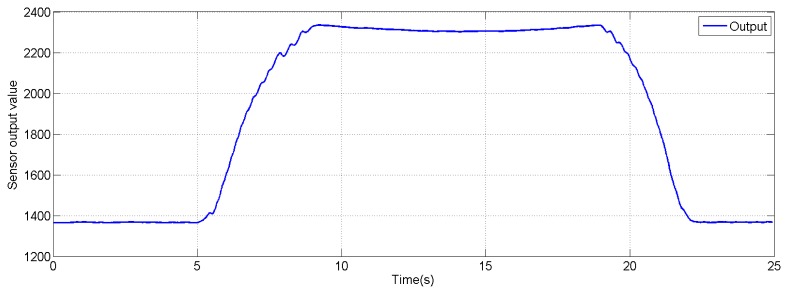
Single sensor output response. Its behavior is purely linear; no hysteresis effect was detected.

The experiments show that the bendable pressure platform is extremely resistant to severe deformation. The design is intended to provide great sensor reliability and repetitiveness. These theoretical features had been experimentally demonstrated. Each pressure sensor of the entire sensor matrix provides a high linear dynamic range behaviour thanks to the signal conditioning electronics; Figure 14 illustrates this response. The illustration correspond to a single sensor response when a 65-kg person is using the pressure platform system (this weight is being distributed over many 20-kg designed sensors placed on the pressure sensor sheet, each sensor output value depending upon the foot position); the lowest sensor output value, 1365 units, is the output value for *V_ref_* ; values greater than *V_ref_* are the pressure detections for a given time, according to the applied pressure.

Each sensor of this bendable pressure matrix is based on the 20-kg maximum detection design, tested in the previous section. The analog voltage measurement range of the embedded electronics is in the range of 0 to 3 V, via a 12-bit Analog to Digital(A/D) converter (4,096 different values). Due to *V_ref_* = 1 V, the effective A/D range is 1 to 3 V, making a theoretical resolution of 2730 units, 7.32 grams per A/D unit. The minimum output value, 1 V, is equivalent to 0 g over the sensor, and the maximum value of Figure 14 is around 2320 sensor units, equivalent to 1.8 V and 10.5 kg over the sensor unit. The embedded electronics firmware provides oversampling and low-pass filtering features in order to minimize raw sensor data disturbances. Outliers are easily eliminated from real-time measured data, resulting in a typical maximum error of 20 grams (an error of 0.1%). This error leads to approximately a third of the theoretical pressure sensing resolution, up to 897 (really, the value is 910, but 13 effective resolution units correspond to the non-recommended 0 to 250 grams zone) different pressure values for these 20-kg designed sensors, which is really not bad compared to commercial applications where the on-screen pressure surfaces use 32 or 64 color palettes (32 or 64 different pressure values); our computer application can use a 897-color palette.

**Figure 15.**
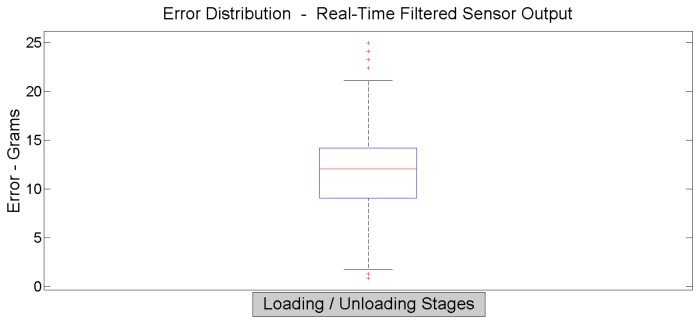
The filtered sensor output error distribution.

Figure 15 shows the error distribution when data acquisition uses oversampling and low-pass filtering, for both load and unload operations in a 1000 iteration test. This recent test is done using a single sensor. The automatic PLC characterization machine does not allow the test over a sensor matrix, due to severe limitations on the available surface.

**Figure 16.**
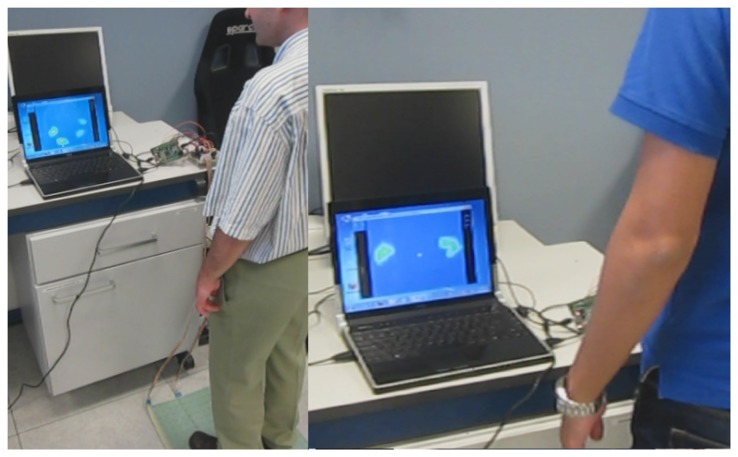
Some users are interacting with the pressure platform system.

The usage of the embedded electronics, the connection to the pressure sensor sheet and the connection to all the system via USB to a PC-style computer is done in a totally plug and play way. The entire system, the pressure platform sheet, the embedded control electronics and the signal conditioning electronics require an electric power source of +5 V and 153 mA, a total power consumption of only 765 mW. The required electric power can be provided directly by the USB port of the computer, the same port used for data exchange. This measure does not take into account the power consumption of the PC computer. Figure 16 shows some users interacting with the pressure platform system.

## 5. Conclusions

Experimental results show a satisfactory accomplishment of the required objectives. These goals are the development and testing of an entirely new type of low-cost and easy to manufacture, bendable pressure sensors, easier electronics integration and the entire system validation, due to the usage as a medical human balance study system, intended for an end-user-like environment (non-typical laboratory scenario). Thanks to the feedback of third party podologists, the end-user application needs to provide more specialized information to be useful for balance study and training purposes. Further work is needed in the end-user application development; also, the spatial resolution needs to be minimized down to 1 cm^2^ instead of the actual 2 cm^2^ size.

The pressure sensor response is almost hysteresis free; however, some signal conditioning and digital processing is needed to provide a satisfactory sensitivity. All tests were made for a 20-kg (per sensor unit) designed pressure sensor and a single electroactive coating composition. More research about different sensor types and electroactive coating compositions needs to be made in the near future. The pressure sensor sheet, a large pressure sensor matrix (256 sensors), presents great difficulties in being used for large iterative tests, due to its large size.

**Figure 17.**
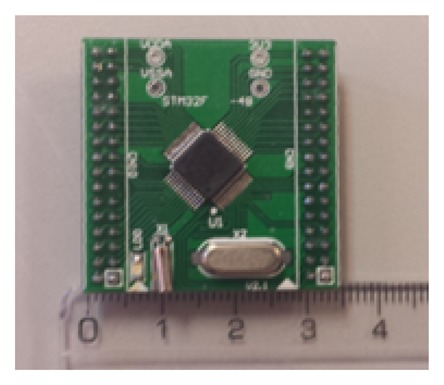
Reduced RCP system to miniaturize the size of the electronics. The units are centimeters.

The usage of the RCP system developed at UC3M RoboticsLab proves to be a great decision, due to the quick integration of the data acquisition and processing stages; thanks to the huge quantity of embedded digital and analog interfaces, as well as to the great computational resources. Extremely low electric power is required for the entire system power. It is entirely provided by an USB port. The RCP system is a low-cost electronics integration technology, which costs around $15 (USD). Due to the overnumbered I/O ports of the common RCP board, a miniaturized version was developed. It can be seen in Figure 17, providing the required analog input ports and the same digital output interfaces: USB, CAN Bus and UART, using a MCU of the same family (STM32F4).
